# The abnormal expression of oxytocin receptors in the uterine junctional zone in women with endometriosis

**DOI:** 10.1186/s12958-016-0220-7

**Published:** 2017-01-03

**Authors:** Miaomaio Huang, Xuqing Li, Peipei Guo, Zhaojuan Yu, Yuting Xu, Zhaolian Wei

**Affiliations:** 1 Department of Gynecology and Obstetrics, First Affiliated Hospital of Anhui Medical University, Meishan Road, Hefei, 230000 China; 2 Assisted Reproductive Center, First Affiliated Hospital of Anhui Medical University, Meishan Road, Hefei, 230000 China

**Keywords:** Endometriosis, Oxytocin receptor, Junctional zone

## Abstract

**Background:**

The junctional zone (JZ), also called as the endometrial-myometrial junction, is related to peristaltic-like movements in the non-pregnant uterus. Hyperperistalsis and dysperistalsis of uterus constructions might underlie many important disorders such as dysmenorrhea, infertility, endometriosis, implantation failure. The major proteins for uterine contraction of the non-pregnant uterus may be Oxytocin (OT) and oxytocin receptor (OTR). The objective of this study was to inspect the expression of OTR in isthmic and mid-fundal parts of the uterine junctional zone at different stages of the follicular cycle in patients with and without endometriosis.

**Methods:**

Uterine biopsies containing endometrium and junctional zone were collected from the isthmic and mid-fundal parts of the anterior wall after hysterectomy. The OTR expression was evaluated by immunohistochemistry.

**Results:**

In the control uterus, OTR expression in the isthmic region was significantly higher than in the fundal region in the proliferative phase (*p* < 0.05) but significantly lower in the secretory phase (*p* < 0.05). And the expression of OTR in the proliferative phase was significantly higher than that in the secretory phase in both isthmic and fundal regions (*p* = 0.000 and 0.049, respectively). However, in endometriosis uteri, OTR expression in the isthmic region showed no significant difference with that in the fundal region in both proliferative and secretory phases (*p* = 0.597 and 0.736, respectively). In both isthmic and fundal regions, OTR expression was not significantly different between the proliferative phase and secretory phase (*p* = 0.084 and 0.222, respectively). OTR expression in fundal regions of revised ASRM I and II endometriosis were lower than that of revised ASRM III and IV (*p* = 0.049). In the fundal region of JZ, the expression of OTR in ovarian endometriosis was significantly lower than that in deep infiltrating endometriosis (*p* = 0.046). The expression level of OTR in the funds region is positively associated with the severity of dysmenorrhea in endometriosis group (*r* = 0.870, *p* < 0.05). Comparing to normal uteri, the expression of OTR in the secretory phase was significantly higher in the endometriosis uteri (*p* < 0.05). In the fundus of endometriosis uteri, OTR expression was significantly higher in both the proliferative and secretory phases (*p* = 0.045 and 0.028, respectively).

**Conclusion:**

OTR expression in the JZ of women with endometriosis changes significantly, which may result in abnormal uterine contractile activity, reducing the endometriosis-related fertility and dysmenorrhea.

## Background

Endometriosis is a chronic disease characterized by the abnormal growth of the endometrial gland and stroma are present in locations other than the lining of the uterus [1, 2]. The JZ, also known as the inner myometrium, is the transitional interface between the endometrium and the outer myometrium [3]. It is not only functionally but also structurally different from the outer myometrium. In the non-gravid uterus, myometrial contractions exclusively distribute from this layer and their amplitude, frequency, and orientation are correlated with menstrual cycle phase [4–6]. These uterine contractions play vital roles in several reproductive processes, including of menstrual flow regulation, rapid sperm transport, successful implantation and deep placentation [5, 7–9]. The JZ seemingly gets involved in the process that determines endometriosis, infertility or pelvic pain. There was a study demonstrated the JZ contractions of isthmic in the proliferative phase were responsible for rapid sperm transport [9]. As for characterization of JZ, the three-dimensional transvaginal sonography can prove to be an accurate diagnostic tool that can be performed easily and repeatedly in patients with endometriosis in lower and advanced stages. The maximum thickness and alteration of JZ in women with endometriosis are significantly greater than those in women without endometriosis [10]. Endometriosis is often associated with JZ alteration even in the minimal and mild stages. JZ alteration could be considered as a beneficial and indirect sign to make a minimally invasive diagnosis of the disease. Thus, an accurate and analytical evaluation of JZ and its potential modification provides important information for women with endometriosis [11].

It is agreed that oxytocin, which can significantly increase the frequency of uterus peristaltic contractions, is one of the most vital mediators for regulating the contraction of uterus, not only in pregnancy but also during the non-pregnant states [5, 12, 13]. Oxytocin receptor is widely expressed in epithelial cells and smooth muscle cells of human uterus and peritoneal endometriotic lesions and ovarian endometriotic cysts [14]. Real time ultrasound has demonstrated peristaltic-like movements which are oxytocin-dependent are confined to the endometrium and JZ in non-pregnant uterus [5, 9]. According to the phases of menstrual cycle, many studies have shown the change of the amplitude, frequency and direction of the contractions of the junctional zone in normal uterus and uterus with endometriosis [5, 7, 8, 15]. Compared to the control, women with endometriosis showed an obvious uterine hyperperistalsis and dysperistalsis [15]. We previously showed the higher serum oxytocin level and higher frequency uterine contractions in endometriotic patients [16]. Abnormal uterine contractile activity might underlie important disorders such as dysmenorrhea, infertility, endometriosis, implantation failure, spontaneous miscarriage or preterm birth [17]. Strong evidence proves that uterus hyperperistalsis is significantly related to the progression of endometriosis [18]. To date, our knowledge is incomplete about the pathophysiologic mechanism governing eccentric contractile activity in the junctional zone in women of endometriosis. As far as we know OTR expression in the isthmus and fundus of JZ of at different menstrual phases in endometriosistic women has not been studied. Therefore, to understand the pathophysiological mechanisms governing abnormal uterine contractions surveyed in endometriosis, we inspected the OTR expression in isthmic and mid-fundal parts of the uterus at different stages of the follicular cycle in patients with and without endometriosis.

## Methods

### Subjects

The study was approved by the Ethics Committee of the First Affiliated Hospital of Medical University of Anhui (reference number: PJ20160409). There were two groups of patients with and without endometriosis. The mean age of patients in the endometriosis and control groups was 42.3 ± 3.9 and 41.5 ± 4.1 years old, respectively. The inclusion criteria for women with endometriosis were who had regular menstrual cycles (23–35 days), histologically confirmed endometriosis, experienced dysmenorrhea or not, received no hormone therapy or used an intrauterine device ≥ six months before hysterectomy. The exclusion criteria for study group were menopausal, adenomyosis, pelvic inflammatory disease and pregnancy 3 months before hysterectomy. Endometriosis and adenomyosis came to be seen as distinct entities. MRI is the method of choice for imaging and evaluation of JZ as an important diagnostic marker in the diagnosis of adenomyosis [19]. Because adenomyosis can be confidently diagnosed using MRI when the altered junctional zone thickness is greater than 12 mm and the expression pattern of oxytocin receptor in the junctional zone in women with adenomyosis had been studied by Zhang, women with evidence of adenomyosis were excluded from the scope of this study [20, 21]. The strict inclusion and exclusion criteria can insure the validity of causal relationship of the findings with the endometriosis against the probably confounder factors exist in our study. The control group included women with regular menstrual cycles who underwent hysterectomy due to cervical intraepithelial neoplasia III (CIN III) or stage I cervical cancer, but with no history of primary dysmenorrhea, no evidence of endometriosis and adenomyosis through gynecological and sonographic examination before surgery, no hormone therapy or use of an intrauterine device ≥ six months before surgery. There were no evidences of endometriosis and adenomyoisis through surgical examination and histology after surgery. According with the principle of ethics, all samples containing endometrium and junctional zone must from the excised uterus. There was no evidence that OTR expression in smooth muscle cells of CIN III or stage I cervical cancer changed comparing with normal uterus. In many studies about OTR expression in smooth muscle cells, CIN III or stage I cervical cancer patients were listed as control groups [13, 20]. Therefore, we incline to the view that OTR expression in JZ of CIN III or stage I cervical cancer patients have no changed compare with normal uterus. Based on the endometrial histology, there were 21 women with endometriosis, 15 women in the proliferative phase and 6 women in the secretory phase, as well as 48 women without endometriosis, with 26 women in the proliferative phase and 22 women in the secretory phase. In the control group, 9 patients suffered from secondary dysmenorrhea due to uterine leiomyoma. Dysmenorrhea is one of the most prevalent symptoms of uterine leiomyoma. High expression of myostatin and matrix- metalloproteinases-14 in uterine leiomyoma correlate with the presence of svere dysmenorrhea [22]. In many studies about OTR expression in smooth muscle cells, uterine leiomyoma patients were listed as control groups [13, 23]. Staging of endometriosis was performed according to the revised classification of the American Society of Reproductive Medicine (revised ASRM: I = 4; II = 2; III = 8, IV = 7). Seventeen ovarian endometriosis (OEM) and four deep infiltrating endometriosis (DIE) patients were in the study group. The severity of the recent dysmenorrhea in the endometriosis group was evaluated by a 10-cm Visual Analog Scale (VAS) before the surgery. The characteristics of the recruited patients of the case and control groups are listed in Table 1.

**Table 1:** Characteristics of the recruited patients with and without endometriosis

Variable	Study group (*n* = 21)	Control group (*n* = 48)	*P* value
Age (in year; mean ± SD)	42.3 ± 3.9	41.5 ± 4.1	.419
Menstrual phase			
Proliferative	15	26	.179
Secretory	6	22	
Indications for hysterectomy			NA
Cervical cancer	2^a^	36	
Ovarian endometriosis	15	0	
Deep infiltrating endometriosis	4	0	
CIN III	0	12	
History of dysmenorrhea			
No	0 (0%)	39 (81.3%)	.000
Yes	21 (100%)	9 (18.7%)	

*NA* not applicable

^a^ovarian endometriosis

### Histological specimens

The uterine junctional zone was defined as the inner third of the myometrium [24]. All samples containing endometrium and junctional zone were collected from the mid-fundal and isthmic areas of the uterine anterior wall. The uterus was opened along the sagittal plane after surgery. Multiple 1 × 1 × 1 cm^3^ samples were collected and fixed in buffered formaldehyde and processed routinely for paraffin embedding. Serial 4 μm sections were prepared from each paraffin-embedded tissue block and handled for immunohistochemical staining. All uterine samples were examined by optical microscope to confirm presence of endometriosis, absence of adenomyosis and respective phases of the menstrual cycle. Under low light microscopy magnification, the uterine junctional zone was located just 3 mm below the endometrium [20].

After routine de-paraffinization and rehydration procedures, the slides were heated in a microwave oven (700 W) in citrate buffer saline (9.0) for twelve min and cooled at room temperature for antigen retrieval. Every sections were incubated with a drop of 3% H_2_O_2_ deionized water (PV-6000, Wuxi, China) for 20 min at 37 °C temperature. After 2 washes with phosphate-buffered saline (PBS), the slides were hatched with polyclonal rabbit anti-OTR (1:100 dilution, bs-1314R, Bioss, Beijing, China) overnight at 4 °C refrigerator. After 3 washes with phosphate-buffered saline (PBS), the sections were incubated with biotinylated anti-rabbit immunoglobulin G (1:400) for 30 min at room temperature. The bound antibody complexes were stained for 3 mins with diaminobenzidine. The slides were then washed, counterstained with hematoxylin, dried and mounted. Negative control sections were processed by omitting the primary antibody. Myometrium of pregnant uterus were used as positive controls. Immunoreactivity staining was characterized quantitatively by digital image analysis on the Image Pro-Plus 6.0 (Nikon, Japan). Images were obtained with a microscope fitted with a digital camera. A series of 4 random images on several sections were taken for each immunostained parameter to obtain a mean value. Staining was defined by color intensity, and a color mask was made. The mask was then applied equally to all images, and measurements were obtained. Immunohistochemical parameters were assessed in the area detected by total optical density and mean optical density, which is equivalent to the intensity of staining in the positive cells.

### Statistical analysis

The results were presented as mean ± standard error of the mean. Statistical Program for Social Sciences (SPSS) for windows version 16.0 (IBM Corp, Armonk, NY, USA) was used to perform statistical analysis. Statistical comparison of data was carried out by Student’s *t*-test for non-paired samples. The normality tests showed that all data were in normal distribution. To evaluate possible effect of OTR expression levels on VAS score, a linear regression model was used. *P*-value less than <0.05 (*p* < 0.05) were considered statistically significant.

## Results

**Fig. 1:**
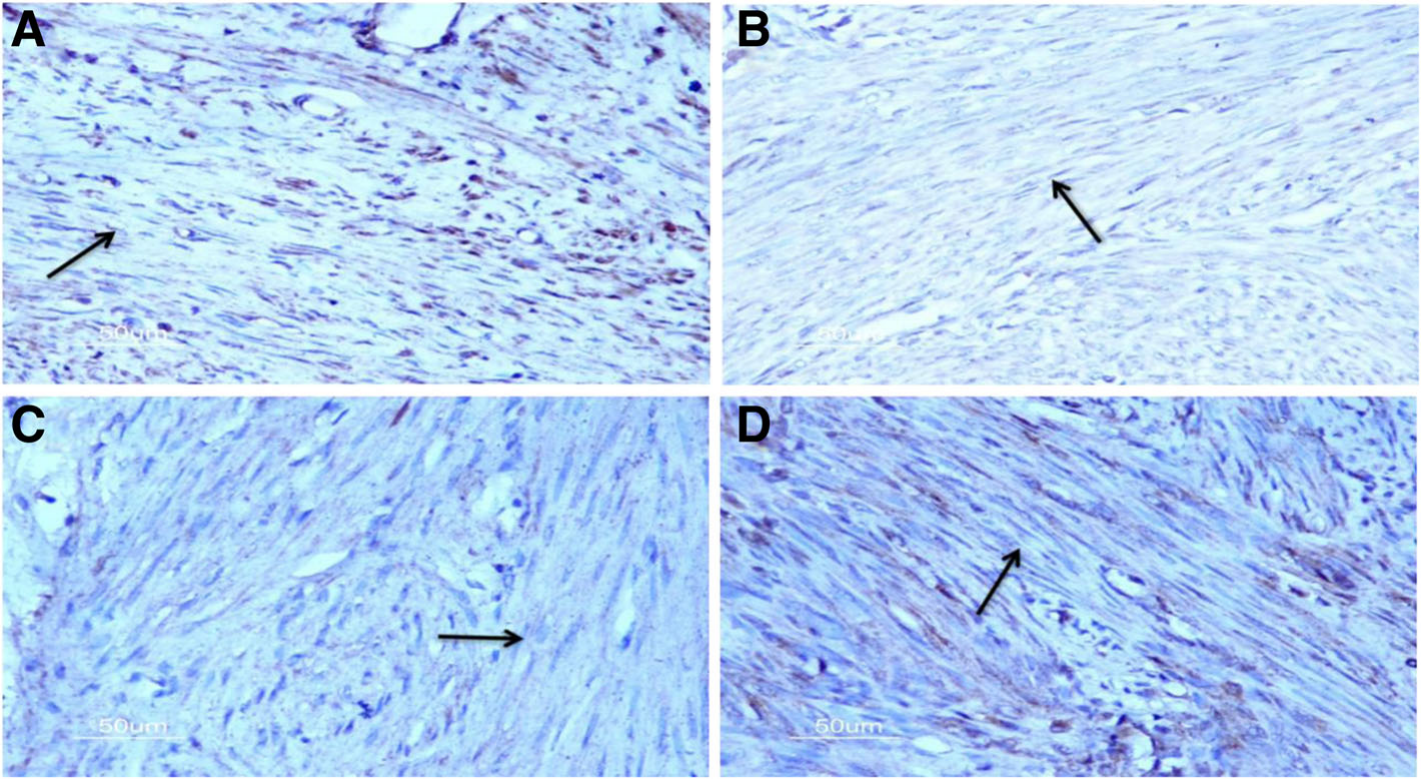
Representative staining of mild cytoplasmic OTR expression in myometrial cells of JZ in the control group (*arrow*). **a** Isthmus region in the proliferative phase. **b** Fundus in the proliferative phase. **c** Isthmus region in the secretory phase. **d** Fundus region in the secretory phase

**Fig. 2:**
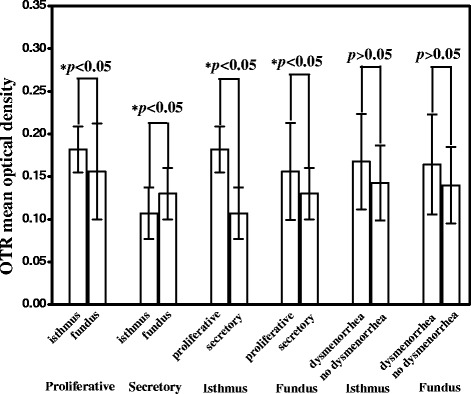
Comparisons of oxytocin expression level in the isthmus and fundus of control uterus in different menstrual cycles. OTR expression in the isthmic region was significantly higher than in the fundal region in the proliferative phase (*p* < 0.05) but significantly lower in the secretory phase (*p* < 0.05). In both isthmic and fundal regions, OTR expression in the proliferative phase was significantly higher than that in the secretory phase (*p* < 0.05). OTR expression in patients with dysmenorrhea of control group was no significant difference from that of patients without in both isthmic and fundal regions of JZ (*p* > 0.05). Data are expressed as mean ± standard error of the mean

The staining of OTR expression in the control uterus is showed in Fig. 1 and the quantitation of OTR expression in Fig. 2. OTR expression in the isthmic region of JZ was significantly higher than in the fundal region in the proliferative phase (*p* = 0.048) but significantly lower in the secretory phase (*p* = 0.012). In both isthmic and fundal regions of JZ, OTR expression in the proliferative phase was significantly higher than that in the secretory phase (*p* = 0.000 and 0.049, respectively). OTR expression in patients with dysmenorrhea of control group was no significant difference from that of patients without in both isthmic and fundal regions of JZ (*p* = 0.154 and 0.175, respectively).

**Fig. 3:**
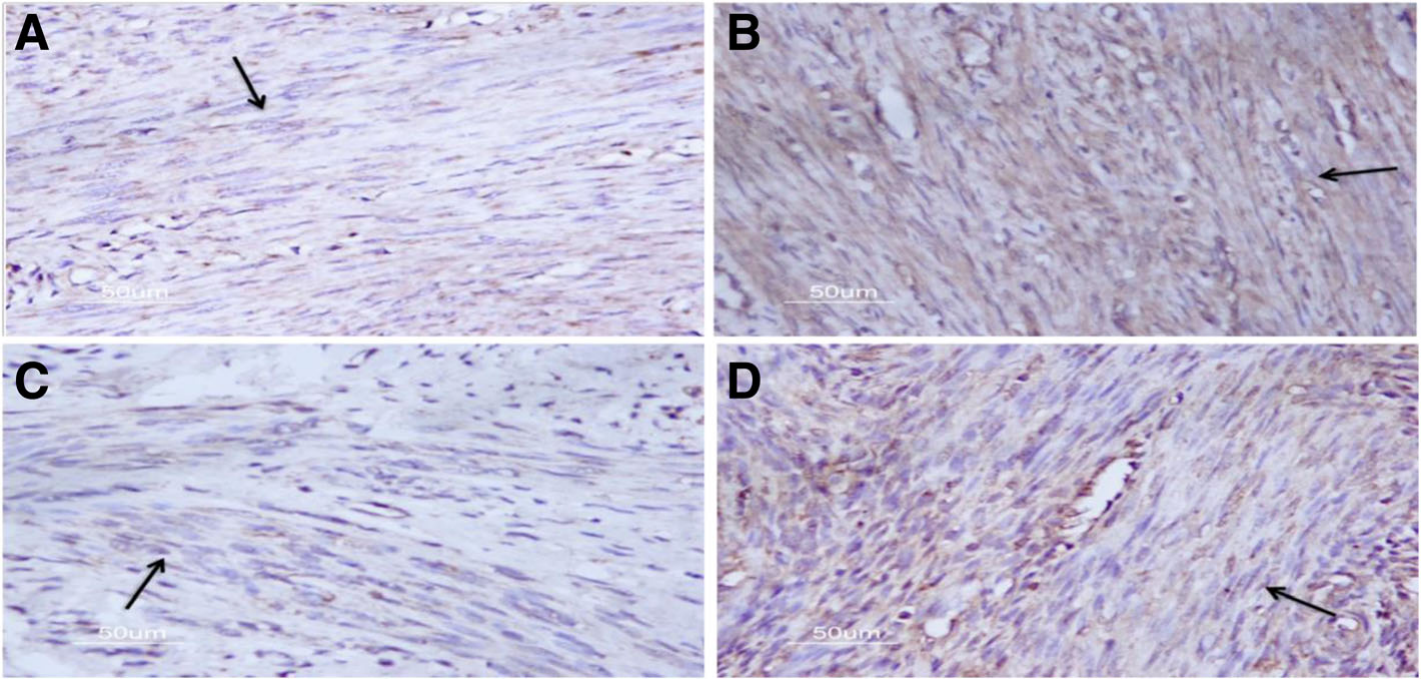
Representative staining of strong cytoplasmic OTR expression in myometrial cells of JZ in the endometriosis group (*arrow*). **a** Isthmus region in the proliferative phase. **b** Fundus in the proliferative phase. **c** Isthmus region in the secretory phase. **d** Fundus region in the secretory phase

**Fig. 4:**
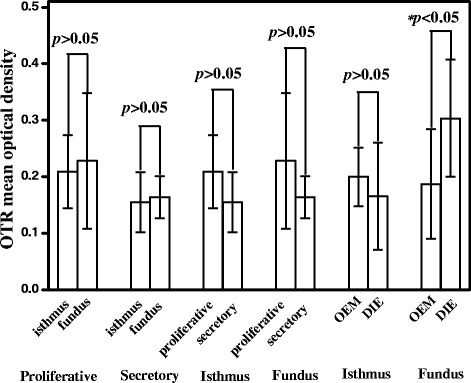
Comparisons of oxytocin expression level in the isthmus and fundus of endometriosis uterus in different menstrual cycles. OTR expression was not significantly between the isthmic and fundal regions in both proliferative and secretory phases. (*p* > 0.05). In both isthmic and fundal regions, the expression of OTR in the proliferative phase was not significantly different from secretory phase (*p* > 0.05). In the fundal region of JZ, the expression of OTR in ovarian endometriosis was significantly lower than that in deep infiltrating endometriosis (*p* < 0.05). Data are expressed as mean ± standard error of the mean

The staining and quantitation of OTR expression of JZ in the endometriosis uteri are showed in Figs. 3 and 4, respectively. OTR expression in the isthmic region was slightly, but not significantly, lower than in the fundal region in both proliferative and secretory phases (*p* = 0.597 and 0.736, respectivley). In both isthmic and fundal regions, the expression of OTR was not significantly different between the proliferative phase and secretory phase (*p* = 0.084 and 0.222, respectively). In the fundal region of JZ, the OTR expression was no significant difference between rASRM I and rASRM II stages of endometriosis (*p* = 0.919). Similarly, there was no significantly different of OTR expression in fundal part of JZ between rASRM III and IV stages of endometriosis (*p* = 0.445). However, OTR expression in fundal regions of revised ASRM I and II endometriosis were lower than that of revised ASRM III and IV (*p* = 0.049). The OTR expression in the isthmic region of JZ in ovarian endometriosis was no significantly different from that of deep infiltrating endometriosis (*p* = 0.357). In the fundal region of JZ, the expression of OTR in ovarian endometriosis was significantly lower than that in deep infiltrating endometriosis (*p* = 0.046).

**Fig. 5:**
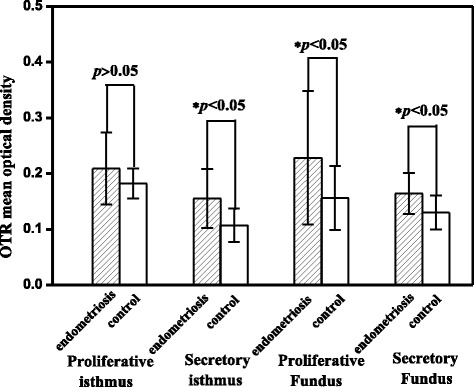
Comparisons of oxytocin receptor expression level between control and endometriosis uteri in the proliferative and secretory phases. In the isthmic region, the expression of OTR in the secretory phase was significantly higher in the endometriosis uteri (*p* < 0.05). In the fundal region of endometriosis uteri, the expression of OTR was significantly higher than that in the control uteri in both the proliferative and the secretory phases (*p* < 0.05). Data are expresses as mean ± standard error of the mean

In the isthmic region, the expression of OTR in the endometriosis uteri showed no significant difference with that in control uterus in the proliferative phase (*p* = 0.139). However, the expression of OTR in the secretory phase was significantly higher in the endometriosis uteri (*p* = 0.007). In the fundal region of endometriosis uteri, the expression of OTR was significantly higher than that in the control uteri in not only the proliferative but also the secretory phases (*p* = 0.045 and 0.028, respectively) Fig. 5.

**Fig. 6:**
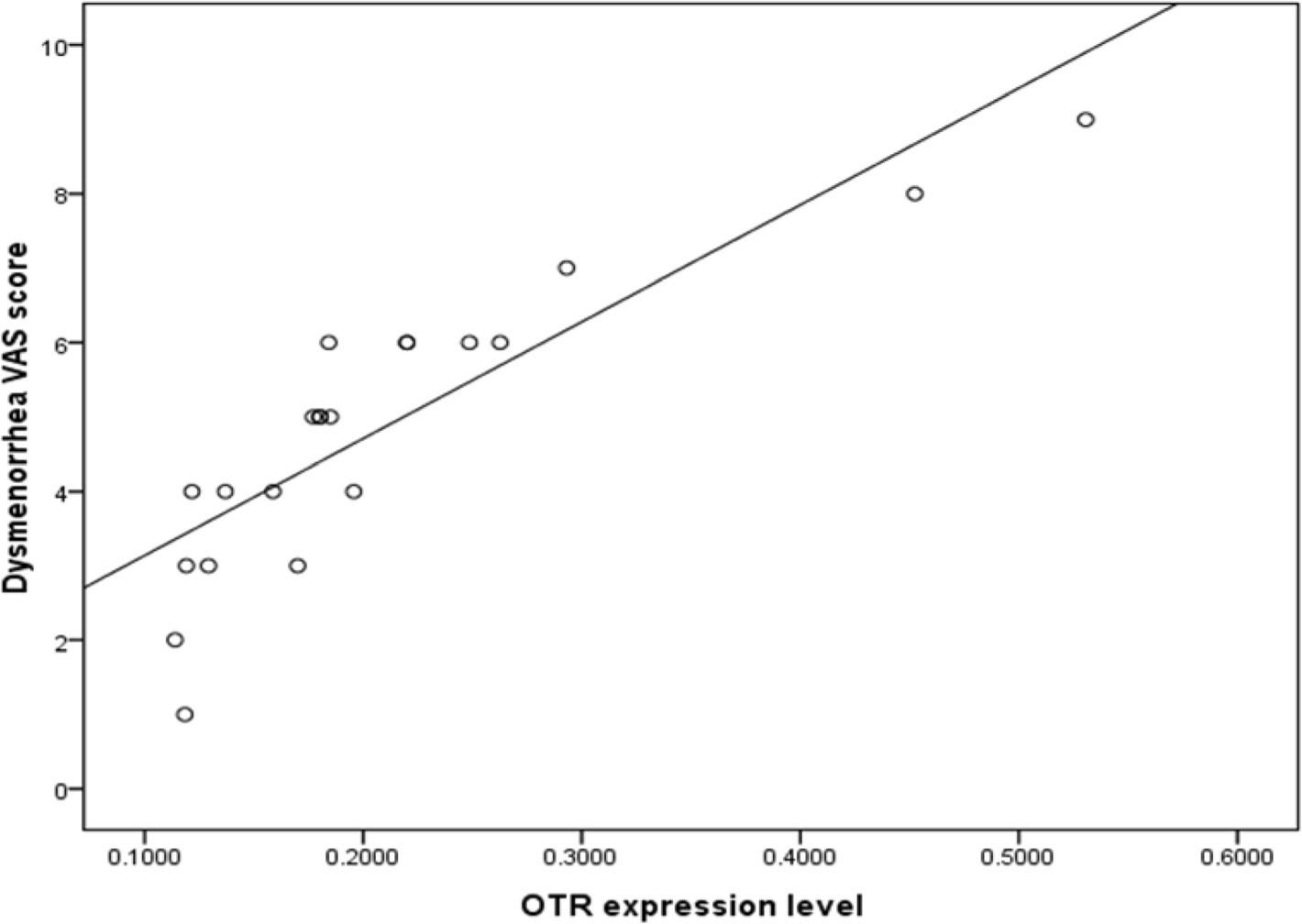
The severity of dysmenorrhea measured by the VAS as function of the OTR expression levels in women with endometriosis. OTR expression level in the funds region is positively correlated with the severity of dysmenorrhea (*r* = 0.872, *p* < 0.05)

We have also found that OTR expression level in the funds region is positively correlated with the severity of dysmenorrhea in women with endometriosis (*r* = 0.870, *p* < 0.05, Fig. 6).

## Discussion

The temporal and spatial OTR distribution in the normal uterus would both change during the menstrual cycle, which are consistent with Zhang et al. [20]. The elevation of OTR expression in the isthmic is to induce an increase uterine contractility of the isthmic part in the proliferative phase. As reported, the velocity and frequency of uterine contractions were maximized at the pre-ovulatory phase of the follicular cycle. They also surveyed that constructions of cervix to fundal pattern were dominant at this phase of the follicular cycle [25–28]. This type of uterine contractions, which aspirate sperm into the cervical part and the uterine cavity, controls the rapid sustained sperm transport and provides forces into the isthmic part of the tubes where they are available for fertilization [29]. In contrast, OTRs were significantly lower in the isthmic part in the secretory phase. The uterine contraction wave at the luteal phase consists of cervico-fundal contractions and isthmus contractions [30]. Both types of uterine peristaltic activities become less frequent by the end of the menstrual cycle. For each contraction started in the isthmical region, only one fourth reaches the fundal region in the late luteal phase. This means the contractions render the fundal region with relative peristaltic quiescence during the late luteal phase and minimizes the mechanical irritation of embryo implantation.

In uterine myometrial cells, OTR expressions are induced by estradiol stimulation and down-regulated by progesterone application [31–34]. OTR concentrations are higher during the proliferative phase than the secretory phase [35–37]. Consistent with these findings, our study revealed OTR expression of control uteri in the proliferative phase showed higher significance difference compared with that in the secretory phase in both isthmic region and fundal region, presumably reacting the positive and negative effects of estrogen and progesterone activity on oxytocin receptor synthesis and degradation respectively [31, 38]. 17β-estradiol (E2), which can be locally secreted by endometriotic cells, plays a vital role in development and progression of endometriosis [39]. The OTR distribution in endometriosis uteri is altered by excessive estrogen. OTR expression was not significantly different between the isthmic and fundal regions at either proliferative or secretory phases. This may be one reason why endometriosis had an obvious uterine hyperperistalsis and dysperistalsis, which prejudice the forces for rapid and sustained sperm transport into tube ipsilateral to the dominant follicle [12, 18, 29].

Women with endometriosis display a marked increase in uterine contraction compared with women free of disease [15, 16, 40]. The frequency of peristaltic activity is doubled at the early and mid-menstrual phase of the cycle compared with normal women [15]. The frequency of peristaltic activity is similar to that obtained in normal women with intravenous injection of oxytocin [41]. During menstruation, the frequency, amplitude and incidence of retrograde uterine contractions in women with endometriosis were much higher than in women without endometriosis [42]. Our previously study revealed women with endometriosis displayed higher frequency uterine contract activity. We also found that dysperistalsis uterine contractions referred to some of the contractions originating in the middle portion of the uterus and spreading simultaneously to the fundus and the cervix. Other contractions that started simultaneously at different sites vanished before they had reached the fundal part of the uterus [16]. Hysterosalpingoscintigraphy revealed that in the early and mid-follicular phases, women with endometriosis displayed hyperperistalsis that resulted in a dramatic increase transportation of inert particles from the vagina, through the uterus into the oviduct and also into the peritoneal cavity [15]. Hyperperistalsis results in desquamation of viable fragments of basal endometrium and enhanced trans-tubal dissemination of these fragments. These fragments might implant some certain sites of the peritoneal cavity [18]. These viable endometrial cells ultimately develop into endometriotic implants. Therefore, the alterations of uterine peristaltic activity play a causal role in the pathogenesis of endometriosis. As reported, volumes of endometriotic implants in rats were significantly reduced by treatment with an OTR antagonist. It indicated that an OTR antagonist may be an important effective drug to decrease dissemination and thereby to combat endometriosis [43]. Blocking OTRs for endometriosis treatment may indirectly inhibit the synthesis aromatase enzyme and the controlled hyper-estrogenic state of the implants.

Dysmenorrhea is related to an elevated basal intrauterine pressure tone and alters the frequency, amplitude and duration of uterine contraction [44]. Since uterine contractions of the JZ in non-pregnant uterus are oxytocin dependent, OTR may be involved in dysmenorrhea. Combining with oxytocin, excessive and irregular oxytocin receptor causes higher intrauterine pressure which is related to dysmenorrhea. Our previous study displayed that women with endometriosis showed higher serum oxytocin level [16]. As reported, the uterus contractile amplitude and OTR expression level in myometrial smooth muscle cells were both significantly higher in adenomyosis cases than in controls, and related positively to adenomyosis-associated dysmenorrhea which is connected with increased uterine contractility. Andrographolide which inhibits OTR expression can normalize uterine contractility and pain alleviation [13]. DIE is recognized as the most severe clinical form of endometriosis and it is associated with severe pain symptom [45]. This maybe can explain the OTR expression in fundal region of JZ in DIE endometriosis patients was higher than that of OEM patients. OTR expression change in the myometrial architecture of uteri having adenomyosis support the hypothesis that dysperistalsis plays an essential role in the development of endometriosis and dysmenorrhea [23]. Besides the cause of uterine hyperactivity, OTR expression up-regulates COX-2 and PGF_2α_which is known as a pain mediator [46]. PGF2αcan further increase the level of Oxytocin and PGE_2_ [47–50]. PGF2α Combed with PGE_2_, can cause COX-2 overproduction [51], which in turn increases PGE_2_ synthetize, and thereby causes dysmenorrhea in endometriosis. Similarly, we provide that endometriosis-associated dysmenorrhea correlates positively with OTR expression level in the JZ (Fig. 6). The main limitation of our study was small size. We believe that this result needs future larger, adequately powered investigation.

In normal uterus, uterine contraction frequency and amplitude increased from mid-cycle to maximum values in the late luteal phase. Retrograde uterine activity is most frequent at mid-cycle, which ensures the sperm transport to the distal end of the tubes [28]. In the isthmic region of endometriosis uteri, the expression of OTR showed no significant difference in both the proliferative and secretory phase. At mid-cycle, peristaltic activities in women with endometriosis are replaced by more convulsive uterine activities [15]. This change has a profound effect on the directed sperm transport. Passive sperm transport in the pre-ovulatory is impeded dramatically by uterine dysperistlasis, which induce a reduced aspiration of sperm into the uterine cavity and a seriously impaired directed transport into the tube ipsilateral to the dominant follicle [15]. We assume that the inefficient sperm transport induced by decreased peristalsis may be involved in infertility associated with endometriosis.

This study reveals the expression of OTR in the fundal region in endometriosis uteri was significantly higher compared with normal uteri in both the proliferative and secretory phases. We also displayed that uterine contract activities were positively correlated with serum of oxytocin levels and patients with endometriosis had higher frequency uterine contractions than in the tubal factor group in our former experiments [16, 52]. Thus, we speculated the OT/OTR system in endometriosis may result in hyperperistalsis and poor endometrial receptivity. Uterine peristalsis provides the forces for embryo migration, but adversely affects the likelihood of pregnancy if the peristaltic frequency is too high [53]. It is estimated that uterine contraction at the time of embryo transfer, especially fund-cervical contractile activity, could expel embryos from the uterus [54]. Patients with uterine peristalsis of >3.0 waves/min before embryo transfer had a lower chance of pregnancy compared with those with lower frequencies [55]. In our previous study, uterine contractions were harmful to embryo implantation and women with more uterine contract activities are more likely to be repeated implantation failures [51]. Atosiban, can compete with OT of OTR in uterine smooth muscle cells, decidual cells, and fetal membranes and inhibits OT-induced PGF2a and uterine activities [56]. Some perspective cohort studies show that atosiban may benefit patients who suffer from repeated or once implantation failure undergoing IVF/embryo transfer with cryopreserved embryos [57–59]. Our former study has indicated application of atosiban improved the clinical pregnancy and implantation rates in patients with emdometriosis-associated infertility [16]. Atosiban can relax uterine arteries and improve uterine endometrial receptivity in pregnant rats [60, 61]. One potential mechanism about the beneficial effects of atosiban on implantation and pregnancy is its inhibitory effects on uterine contractions [57].

## Conclusions

The OTR expression in the uterine junctional zone of women with endometriosis appears to have changed significantly and irregularly. The abnormal expression pattern of oxytocin receptor in the JZ in women with endometriosis may result in abnormal uterine contractile activity, reduced fertility and dysmenorrhea associated with endometriosis.

## Abbreviations

CINIII: Cervical intraepithelial neoplasia
JZ: Junctional zone
OT: Oxytocin
OTR: Oxytocin receptor
VIS: Visual analogue scale
